# Long-term Cross-reactivity Against Nonvaccine Human Papillomavirus Types 31 and 45 After 2- or 3-Dose Schedules of the AS04-Adjuvanted Human HPV-16/18 Vaccine

**DOI:** 10.1093/infdis/jiy743

**Published:** 2019-02-03

**Authors:** Nicolas Folschweiller, Ulrich Behre, Marc Dionne, Paolo Durando, Susanna Esposito, Linda Ferguson, Murdo Ferguson, Peter Hillemanns, Shelly A McNeil, Klaus Peters, Brian Ramjattan, Tino F Schwarz, Khuanchai Supparatpinyo, Pemmaraju V Suryakirian, Michel Janssens, Philippe Moris, Annabelle Decreux, Sylviane Poncelet, Frank Struyf

**Affiliations:** 1 GSK, Wavre, Belgium; 3 MedizinischeHochschule Hannover; Hannover; 5 Institute of Laboratory Medicine and Vaccination Centre, Klinikum Würzburg Mitte, Standort Juliusspital, Würzburg, Germany; 6 Centre Hospitalier Universitaire, Québec; 7 Colchester Research Group, Truro; 8 Canadian Center for Vaccinology, IWK Health Centre and Nova Scotia Health Authority, Dalhousie University, Halifax; 9 First Line Medical Services, St. John’s, Canada; 10 Department of Health Sciences, University of Genoa; 11 IRCCS Ospedale Policlinico AOU San Martino-IST, Genoa; 12 Pediatric Clinic, Department of Surgical and Biomedical Sciences, Università degli Studi di Perugia, Perugia, Italy; 13 Chiang Mai University, Thailand; 14 GSK, Bangalore, India

**Keywords:** AS04-HPV-16/18 vaccine, HPV-31, HPV-45, cross-reactivity, nonvaccine types, immunogenicity

## Abstract

This analysis focused on long-term cross-reactive immunogenicity against nonvaccine human papillomavirus (HPV) types 31 and 45 following 2 doses of AS04-adjuvanted HPV-16/18 vaccine in girls aged 9–14 years or following 3 doses in women aged 15–25 years, for up to 3 years (HPV-070 study) and up to 5 years (HPV-048 study) after the first vaccination. Both schedules elicited antibodies against HPV-31 and HPV-45 up to 5 years after first dose. The antibody concentration was similar in young girls as compared to women. Specific CD4^+^ T-cell and B-cell responses to HPV-31 and HPV-45 at month 36 were similar across groups. Clinical trials registration: NCT01381575 and NCT00541970.

## Teaser

The AS04-adjuvanted human papillomavirus type 16/18 (HPV-16/18) vaccine showed sustained cross-reactivity against nonvaccine HPV types 31 and 45 for up to 5 years, whether administered as a 2-dose schedule in girls aged 9–14 years or as a 3-dose schedule in women aged 15–25 years.

The AS04-adjuvanted human papillomavirus (HPV) types 16 and 18 vaccine (hereafter, “AS04–HPV-16/18”) was developed and initially licensed for delivery on a 3-dose schedule, at months 0, 1, and 6. AS04 is an adjuvant system containing 3-O-desacyl-4’-monophosphoryl lipid A (50 μg; GSK) adsorbed on aluminum salt (500 μg Al^3+^). The high immunogenicity of the vaccine observed after 3 doses in girls aged 9–14 years led to the investigation and eventual licensure of a 2-dose schedule, at months 0 and 6, in this age group. The aim of the exploratory phase 1/2 HPV-048 study (clinical trials registration NCT00541970) was to investigate alternative schedules of AS04–HPV-16/18 in subjects aged 9–25 years [1]. The confirmatory phase 3 HPV-070 study (clinical trials registration NCT01381575) aimed at demonstrating the noninferiority of the immune response of the 2-dose schedule (at months 0 and 6) in young girls (age, 9–14 years) versus the 3-dose schedule (at months 0, 1, and 6) in young women (age, 15–25 years) [2], the age group in which AS04–HPV-16/18 efficacy against cervical intraepithelial neoplasia grade 3 or greater was previously demonstrated [3].

Even if HPV-16 and HPV-18, detected in 70% of cases, are the most common types associated with cervical cancer [4], HPV-31 and HPV-45 are also high-risk HPV types, leading to approximately 10% of all cervical cancer cases [5]. Efficacy and immunogenicity studies with 3 doses of AS04–HPV-16/18 have shown a degree of protection (cross-protection) [3, 6] and immunogenicity (cross-reactivity) [7] against nonvaccine HPV types, including HPV-31 and HPV-45. Recent evidence provided by follow-up studies showed long-term cross-protective effectiveness after 3 doses [8–11]. In addition to the vaccine types (ie, HPV-16/18), the HPV-070 and HPV-048 studies also investigated the cellular and humoral immunogenicity of AS04–HPV-16/18 against the nonvaccine HPV types 31 and 45 after 2 doses of the vaccine. Here, we disclose HPV-31 and HPV-45 immunogenicity results from the HPV-048 and HPV-070 studies, along with cellular immune responses from the HPV-070 study.

## METHODS AND PARTICIPANTS

### Study Design, Participants, and Ethics

The HPV-048 (clinical trials registrationNCT00541970) study was a phase 1/2, partially blinded, controlled, randomized (1:1:1:1), parallel group trial conducted at 21 centers in Canada and Germany between October 2007 and March 2013. Study HPV-070 (clinical trials registration NCT01381575) was a phase 3b, multicenter, open-label, randomized trial conducted in 5 countries (Canada, Germany, Italy, Taiwan, and Thailand) between June 2011 and November 2014.

The methods of these studies have been described and published previously [1, 2]. A subset of subjects from both studies was evaluated for the current analysis. All subjects included in the HPV-048 trial and with samples available at the required time points were selected. The 50 first subjects in each age stratum (9–11, 12–14, 15–19, and 20–25 years) from preselected sites were included in the HPV-070 study. The subjects in the humoral immunity subset were the same as those in the cell-mediated immunity subcohort in the HPV-070 study. Immunogenicity data were assessed up to 5 years after first vaccination in the HPV-048 study and for up to 3 years after first vaccination, along with cellular immune responses, in the HPV-070 study.

Both primary studies were approved by an appropriate independent ethics committee or institutional review board and conducted according to the principles of the Declaration of Helsinki, good clinical practice guidelines, and all other regulatory requirements. Written informed consent was obtained from all participants and/or parents or legal representatives prior the start of the study.

### Immunogenicity Assessments

Blood samples for antibody determination were collected from all subjects before the first vaccination (at month 0); at months 7, 12, 18, 24, and 36; and at months 48 and 60 (in the HPV-048 study only). Antibodies to HPV-31 and HPV-45 were assessed by an enzyme-linked immunosorbent assay (ELISA) as previously described [7]. Seroconversion was defined as a serum antibody titer greater than or equal to the cutoff of 59 ELISA units/mL among subjects seronegative to HPV-31 and HPV-45 before vaccination.

In the HPV-070 study, CD4^+^ T-cell responses specific to HPV-31 and HPV-45 were determined by intracellular cytokine staining, as described elsewhere [7]. B-cell responses to HPV-31 and HPV-45 were evaluated by a B-cell enzyme-linked immunosorbent spot assay, as previously described [12].

### Statistical Methods

The descriptive primary analyses were performed on the initially seronegative participants in the month 36 (for the HPV-070 study) and month 60 (for the HPV-048 study) according-to-protocol (ATP) cohorts for immunogenicity (ATP-I); secondary immunogenicity analyses based on the total vaccinated cohort (TVC) were performed to complement the ATP analyses. The analysis was exploratory for the HPV-070 study, and testing and analysis were conducted in a post hoc fashion for the HPV-048 study. The ATP-I included all evaluable participants who met all eligibility criteria, who complied with the study procedures, and for whom data concerning immunogenicity end point measures were available.

Geometric mean concentration (GMC) calculations were performed by taking the antilog of the mean of the log concentration transformations. Seroconversion rates for each antigen and GMC were calculated with exact 95% confidence intervals (CIs) before and after vaccination. An arbitrary value of half the cutoff for the GMC calculation was given to antibody concentrations below the assay cutoff.

## RESULTS

### Study Population

In the HPV-048 study, 960 participants received at least 1 vaccine dose and were included in the TVC. Among them, AS04–HPV-16/18 was administered to 157 participants aged 15–25 years (and 82 participants aged 9–14 years) via the standard 3-dose schedule and to 78 participants aged 9–14 years (and 162 participants aged 15–25 years) via the 2-dose schedule. The other participants (n = 481) received a different vaccine formulation. There were 91 participants aged 15–25 years in the 3-dose group and 46 participants aged 9–14 years in the 2-dose group who were included in the month 60 ATP-I; of these, 37 and 33 participants, respectively, were considered for the HPV-31 and HPV-45 subset analysis [1].

Overall, 967 participants in the HPV-070 TVC (93.7%) received AS04–HPV-16/18 and completed the month 36 visit. Among them, 907 (93.8%) were included in the month 36 ATP-I cohort: 401 were aged 15–25 years and in the 3-dose group, and 506 were aged 9–14 years and in the 2-dose group [2]. Of these participants, 92 and 96, respectively, were considered for the HPV-31 and HPV-45 subset analysis

Demographic characteristics and baseline serostatus, stratified by age and study, are shown for both groups in Supplementary Table 1.

### Immune Response to HPV-31 and HPV-45

All seronegative participants from the HPV-048 study and all but 1 (in the 3-dose group) from the HPV-070 study in the ATP-I seroconverted to HPV-31 and HPV-45 one month after the last vaccine dose (at month 7; Supplementary Table 2). At month 60 in the HPV-048 ATP-I cohort, similar proportions of subjects were seropositive for HPV-31 and for HPV-45 among those vaccinated with the 2-dose and 3-dose schedules: about 90% of subjects were seropositive for HPV-31, and 80.0% were seropositive for HPV-45. Similarly, at month 36 in the HPV-070 ATP-I cohort, about 80% of vaccinees were seropositive for HPV-31 and for HPV-45, irrespective of the group.

**Figure 1.**
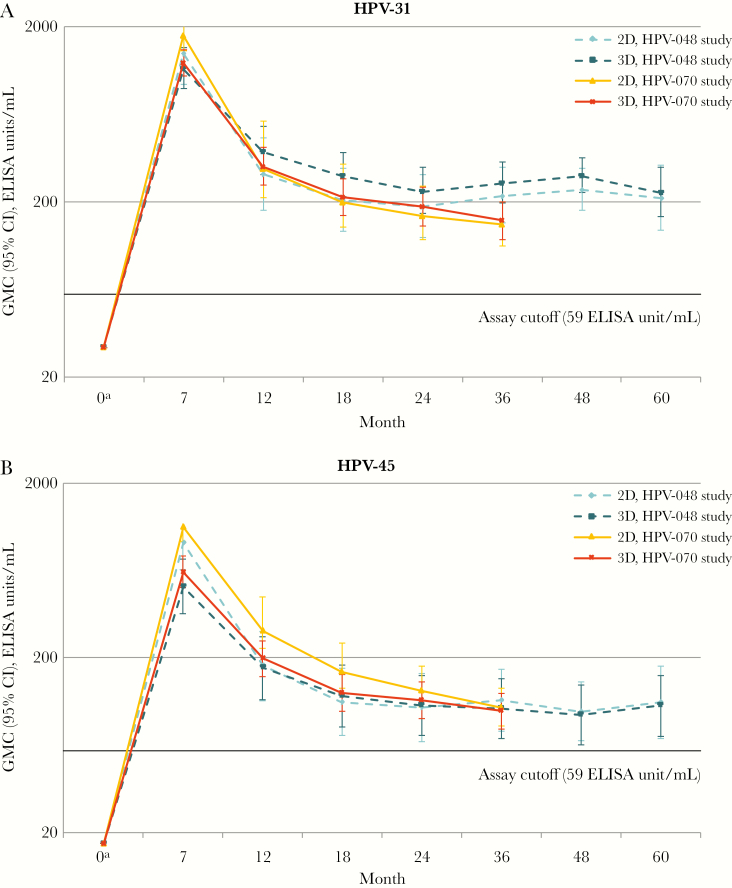
Human papillomavirus type 31 (HPV-31; *A*) and HPV-45 (*B*) geometric mean concentrations (GMCs) by enzyme-linked immunosorbent assay at each time point for initially seronegative subjects in the month 36 (for the HPV-070 study) or month 60 (for the HPV-048 study) according-to-protocol cohort for immunogenicity. CI, confidence interval; 2D, 2-dose schedule of the AS04-adjuvanted HPV-16/18 vaccine; 3D, 3-dose schedule of the AS04-adjuvanted HPV-16/18 vaccine. ^a^The GMC at month 0 was equal to half of the assay cutoff (29.5 ELISA units/mL).

After a peak response at month 7, GMCs of HPV-31 and HPV-45 antibodies started reaching a plateau phase by month 24 in both studies, which was sustained until month 60 in the HPV-048 study (Figure 1). GMCs for HPV-31/45 antibodies were similar in young girls receiving the 2-dose schedule as compared to young adult women receiving the 3-dose schedule (Supplementary Table 3).

Results in the TVC were in line with those observed in the ATP-I (data not shown).

### HPV-31– and HPV-45–Specific B-Cell and T-Cell Responses

**Figure 2.**
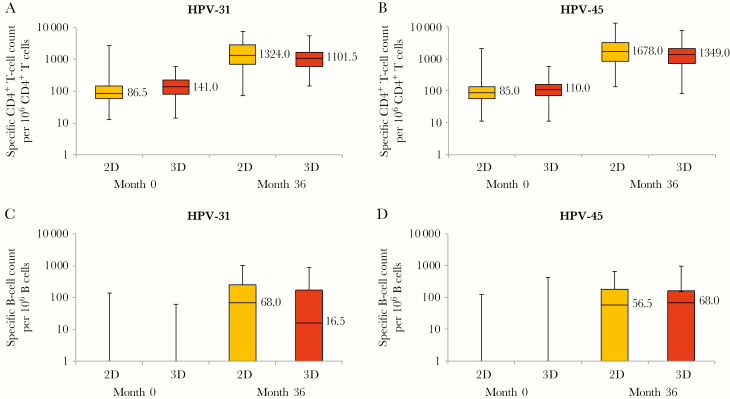
Human papillomavirus type 31 (HPV-31)–specific (*A*) and HPV-45–specific (*B*) CD4^+^ T-cell responses and HPV 31–specific (*C*) and HPV-45–specific (*D*) memory B-cell responses for initially seronegative subjects from the HPV-070 study who were in the month 36 according-to-protocol cohort for immunogenicity. Upper and lower limits of boxes denote interquartile ranges, horizontal lines inside boxes and bars denote median values, and whiskers denote maximum and minimum values. 2D, 2-dose schedule of the AS04-adjuvanted HPV-16/18 vaccine; 3D, 3-dose schedule of the AS04-adjuvanted HPV-16/18 vaccine.

HPV-31– and HPV-45–specific memory B-cell and T-cell responses were detected following vaccination and were similar across groups (Figure 2 and Supplementary Table 4). Similar results as those described above were found in the TVC (data not shown).

## DISCUSSION

In both studies, similar anti–HPV-31 and anti–HPV-45 GMCs and seroconversion rates were observed in young girls receiving the 2-dose schedule as compared to young adult women receiving the 3-dose schedule, with >80.0% of subjects who were initially seronegative seroconverting following vaccination, irrespective of the schedule received. The kinetics of antibodies against nonvaccine HPV types 31 and 45 observed in both trials were similar to that observed against HPV-16 and HPV-18 [2, 13]: there was a peak response after the last vaccine dose, followed by a plateau, with no sign of waning immunogenicity. As expected, GMCs for HPV-31 and HPV-45 appeared to be lower than those for HPV-16 and HPV-18 at similar time points, although GMCs for different types should not be directly compared because they result from separate assays using different arbitrary units (Supplementary Table 5).

HPV-31– and HPV-45–specific CD4^+^ T cells and memory B-cell responses were detected until the end of the HPV-070 study, at month 36, and in similar range across groups. This suggests the ability to produce antibodies against HPV-31 and HPV-45 after challenge or natural infection years after primary vaccination with either a 2-dose or 3-dose schedule. These findings were not surprising, because HPV-31 is closely related to HPV-16 and HPV-45 is closely related to HPV-18. However, in a different study, in which AS04–HPV-16/18 was compared to an aluminum-only adjuvanted HPV vaccine, the amplitude of cross-reactivity was much bigger with the AS04-adjuvanted vaccine. We hypothesize that the adjuvant plays a major role in the production of nonvaccine-type anti-HPV antibodies [7].

Overall, these results provide additional evidence that the cross-protection against HPV-31 and HPV-45 after vaccination with either 2 doses or 3 doses of AS04–HPV-16/18 is likely to be sustained whether the vaccine is given as a 2-dose or a 3-dose schedule.

Our present work has the limitation that no efficacy end points were assessed along with the immunogenicity assessment, owing to the young age of the population studied. However, long-term cross-protection after 3 doses of AS04–HPV-16/18 against HPV-31 and HPV-45, as well as other HPV types, was shown in clinical trials [6] and effectiveness/impact studies [8–11]. Our results suggest that a similar outcome can be expected after 2 doses of the vaccine in young girls.

In summary, cross-reactivity against HPV-31 and HPV-45 is maintained over time and is similar following either 2 doses of AS04–HPV-16/18 in girls or 3 doses in women. The GMC plateau phase observed up to 5 years after vaccination and the presence of memory B cells bring additional evidence that the cross-protection offered by 2 doses of the AS04–HPV-16/18 against nonvaccine types 31 and 45 is long-lasting.

## Supplementary Data

Supplementary materials are available at *The Journal of Infectious Diseases* online. Consisting of data provided by the authors to benefit the reader, the posted materials are not copyedited and are the sole responsibility of the authors, so questions or comments should be addressed to the corresponding author.

jiy743_suppl_Supplementary_Tables_1-5

jiy743_suppl_Supplementary_materials

## Notes

***Acknowledgments.*** The authors thank the study participants and their families for their participation to the HPV-048 or HPV-070 study. They also acknowledge the investigators of the HPV-048 (NCT00541970) and HPV-070 (NCT01381575) trials as well as their clinical teams for their support and care of participants. The authors thank the global and regional clinical teams of GSK for their contribution to the study, the R&D group for testing, processing and coordination of laboratory work, the scientific writers for clinical protocol and clinical report writing as well as the statisticians’ team for input on statistical analysis. The authors would like to thank Business & Decision Life Sciences platform for editorial assistance and publication coordination, on behalf of GSK. Bruno Baudoux coordinated publication development and editorial support. The authors also thank Valérie Patin (Business & Decision Life Sciences, on behalf of GSK) for providing medical writing support.

***Financial support.*** This work was supported by GlaxoSmithKline Biologicals SA.

***Potential conflicts of interest.*** GlaxoSmithKline Biologicals SA supported the HPV-048 and HPV-070 studies and was involved in all stages of study conduct, including analysis of the data. GlaxoSmithKline Biologicals SA also covered all costs associated with the development and publication of this article. N. F., J. M., P. M., A. D., S. P., and F. S. are employees of the GSK group of companies; P. V. S. was an employee of the GSK group of companies at the time the study was conducted; and N. F., P. M., S. P., and F. S. hold shares in the GSK group of companies. P. D. declares that the Department of Health Sciences of the University of Genoa had a contract with the GSK group of companies during the conduct of the HPV-070 trial. S. A. M. received grants for unrelated work from GSK, Pfizer, Sanofi, and Merck; consulting fees from Merck and Pfizer; and honoraria and speaker’s fees from GSK, Merck, Sanofi, and Pfizer for unrelated work. M. D. received a grant from the GSK group of companies through his employer, the CHU of Quebec City, as principal investigator for several GSK clinical trials, including the HPV-048 and HPV-070 studies. S. E. received personal fees from the GSK group of companies, Sanofi Aventis, Merck, and Vifor outside the submitted work and received a grant from the GSK group of companies, Sanofi Aventis, Vifor, Valeas, and DMG outside the submitted work. M. F. received personal fees from the Colchester Research Group (CRG), which is owned by his wife, L. F., in his role as principal investigator/subinvestigator during the conduct of the study; personal fees were also paid from CRG outside the submitted work. T. F. S. received personal fees from the GSK group of companies, Pfizer, and Sanofi Pasteur outside the submitted work. All other authors report no potential conflicts.

Presented in part: 36th Annual Meeting of the European Society for Paediatric Infectious Diseases, Malmö, Sweden, 29 May–2 June 2018.
