# Supplementary material for: Long-term Cross-reactivity Against Nonvaccine Human Papillomavirus Types 31 and 45 After 2- or 3-Dose Schedules of the AS04-Adjuvanted Human HPV-16/18 Vaccine
Source: J Infect Dis. 2019 Feb 3;219(11):1799–803. doi: 10.1093/infdis/jiy743 (PMC6500548; doi:10.1093/infdis/jiy743)
Supplement: jiy743_suppl_Supplementary_Tables_1-5 [file jiy743_suppl_supplementary_tables_1-5.docx]

Supplementary Table 1. Summary of demographic characteristics and baseline serostatus by age stratum and by study (M60 ATP-I for HPV-048 study [NCT00541970] and M36 ATP-I for HPV-070 study [NCT01381575]).

|  | 2D schedule  Girls 9-14 years | | 3D schedule  Women 15-25 years | |  |
| --- | --- | --- | --- | --- | --- |
|  | HPV-048  (N=33) | HPV-070  (N=96) | HPV-048  (N=37) | HPV-070  (N=92) | |
| **Age, y, mean (SD)** | 12.6 (1.6) | 11.5 (1.8) | 19.6 (2.8) | 19.7 (3.0) |  |
| **Geographic ancestry, n (%)** |  |  |  |  |  |
| African heritage / African American | 0 (0.0) | 0 (0.0) | 0 (0.0) | 1 (1.1) |  |
| Asian—Central/South Asian heritage | 0 (0.0) | 0 (0.0) | 0 (0.0) | 0 (0.0) |  |
| Asian—East Asian heritage | 0 (0.0) | 0 (0.0) | 0 (0.0) | 0 (0.0) |  |
| Asian—South East Asian heritage | 0 (0.0) | 20 (20.8) | 0 (0.0) | 17 (18.5) |  |
| White—Arabic / North African heritage | 0 (0.0) | 0 (0.0) | 0 (0.0) | 0 (0.0) |  |
| White—Caucasian / European heritage | 33 (100) | 75 (78.1) | 37 (100) | 73 (79.3) |  |
| Other | 0 (0.0) | 1 (1.0) | 0 (0.0) | 1 (1.1) |  |
| **HPV-31 baseline serostatus, n (%)** |  |  |  |  |  |
| Seropositive^a^ | 0 (0.0) | 0 (0.0) | 0 (0.0) | 0 (0.0) |  |
| **HPV-45 baseline serostatus, n (%)** |  |  |  |  |  |
| Seropositive^a^ | 0 (0.0) | 0 (0.0) | 0 (0.0) | 0 (0.0) |  |

2D, 2-dose schedule (Months 0,6) of the HPV-16/18 AS04-adjuvanted vaccine; 3D, 3-dose schedule (Months 0,1,6) of the HPV-16/18 AS04-adjuvanted vaccine; ATP-I, according-to-protocol cohort for immunogenicity; HPV, human papillomavirus; N, number of subjects in the cohort; n(%), number (percentage) of subjects in the given category; SD, standard deviation. ^a^Seronegative status was defined as an antibody titer lower than the assay cutoff before vaccination (assay cutoff of 59 EU/mL for anti–human papillomavirus [HPV] 31 and for anti–HPV-45).

Supplementary Table 2. Observed HPV-31 and HPV-45 seroconversion rates by ELISA at each time-point for initially seronegative subjects in the M36 (HPV-070; NCT01381575) or M60 (HPV-048; NCT00541970) ATP-I cohort.

| Time-point | Antigen | 2D schedule  Girls 9-14 years | | | 3D schedule  Women 15-25 years | | Antigen | 2D schedule  Girls 9-14 years | | 3D schedule  Women 15-25 years | | |
| --- | --- | --- | --- | --- | --- | --- | --- | --- | --- | --- | --- | --- |
|  |  | N | Seroconversion rate,  % (95% CI) | | N | Seroconversion rate,  % (95% CI) |  | N | Seroconversion rate,  % (95% CI) | N | Seroconversion rate,  % (95% CI) | |
|  | | | | **HPV-048 study** | | | | | | | |  |
| M7 | **HPV-31** | 33 | 100 (89.4; 100) | | 34 | 100 (89.7; 100) | **HPV-45** | 30 | 100 (88.4; 100) | 35 | 100 (90.0; 100) | |
| M12 |  | 33 | 97.0 (84.2; 99.9) | | 34 | 97.1 (84.7; 99.9) |  | 30 | 93.3 (77.9; 99.2) | 35 | 97.1 (85.1; 99.9) | |
| M18 |  | 33 | 84.8 (68.1; 94.9) | | 34 | 97.1 (84.7; 99.9) |  | 30 | 83.3 (65.3; 94.4) | 35 | 85.7 (69.7; 95.2) | |
| M24 |  | 33 | 87.9 (71.8; 96.6) | | 34 | 88.2 (72.5; 96.7) |  | 30 | 83.3 (65.3; 94.4) | 35 | 85.7 (69.7; 95.2) | |
| M36 |  | 32 | 93.8 (79.2; 99.2) | | 34 | 94.1 (80.3; 99.3) |  | 29 | 86.2 (68.3; 96.1) | 35 | 82.9 (66.4; 93.4) | |
| M48 |  | 32 | 93.8 (79.2; 99.2) | | 31 | 93.5 (78.6; 99.2) |  | 29 | 89.7 (72.6; 97.8) | 33 | 81.8 (64.5; 93.0) | |
| M60 |  | 33 | 93.9 (79.8; 99.3) | | 34 | 91.2 (76.3; 98.1) |  | 30 | 80.0 (61.4; 92.3) | 35 | 80.0 (63.1; 91.6) | |
|  | | | | **HPV-070 study** | | | | | | | |  |
| M7 | **HPV-31** | 90 | 100 (96.0; 100) | | 85 | 98.8 (93.6; 100) | **HPV-45** | 91 | 100 (96.0; 100) | 86 | 100 (95.8; 100) | |
| M12 |  | 90 | 95.6 (89.0; 98.8) | | 85 | 94.1 (86.8; 98.1) |  | 91 | 97.8 (92.3; 99.7) | 86 | 93.0 (85.4; 97.4) | |
| M18 |  | 89 | 91.0 (83.1; 96.0) | | 85 | 90.6 (82.3; 95.8) |  | 90 | 96.7 (90.6; 99.3) | 86 | 86.0 (76.9; 92.6) | |
| M24 |  | 90 | 85.6 (76.6; 92.1) | | 85 | 88.2 (79.4; 94.2) |  | 91 | 93.4 (86.2; 97.5) | 86 | 83.7 (74.2; 90.8) | |
| M36 |  | 90 | 82.2 (72.7; 89.5) | | 85 | 85.9 (76.6; 92.5) |  | 91 | 84.6 (75.5; 91.3) | 86 | 82.6 (72.9; 89.9) | |

Seronegative status was defined as an antibody titer lower than the assay cut-off before vaccination (assay cut-off of 59 enzyme-linked immunosorbent assay units [EU]/ml).

2D, 2-dose schedule (M0,6) of the HPV-16/18 AS04-adjuvanted vaccine; 3D, 3-dose schedule (M0,1,6) of the HPV-16/18 AS04-adjuvanted vaccine; ATP-I, according-to-protocol immunogenicity cohort; 95% CI, two-sided 95% confidence intervals; ELISA, enzyme-linked immunosorbent assay; EU, enzyme-linked immunosorbent assay units; HPV, human papillomavirus; M, month; N, number of subjects with pre-vaccination results available.

Supplementary Table 3. Observed HPV-31 and HPV-45 GMC by ELISA at each timepoint for initially seronegative subjects in the M36 (HPV-070; NCT01381575) or M60 (HPV-048; NCT00541970) ATP-I cohort.

| Time-point | Antigen | 2D schedule  Girls 9-14 years | | 3D schedule  Women 15-25 years | | Antigen | 2D schedule  Girls 9-14 years | | 3D schedule  Women 15-25 years | |
| --- | --- | --- | --- | --- | --- | --- | --- | --- | --- | --- |
|  |  | N | GMC, (95% CI) | N | GMC, (95% CI) |  | N | GMC, (95% CI) | N | GMC, (95% CI) |
| **HPV-048 study** | | | | | | | | | | |
| M7 | **HPV-31** | 33 | 1406.0 (1038.8; 1903.0) | 34 | 1153.9 (783.3; 1699.9) | **HPV-45** | 30 | 1551.9 (1078.7; 2232.6) | 35 | 865.2 (603.5; 1240.3) |
| M12 |  | 33 | 290.0 (205.6; 409.1) | 34 | 387.2 (254.5; 589.0) |  | 30 | 305.0 (193.8; 480.0) | 35 | 297.6 (196.7; 450.2) |
| M18 |  | 33 | 204.5 (141.3; 296.0) | 34 | 281.7 (197.7; 401.5) |  | 30 | 190.2 (122.1; 296.1) | 35 | 204.8 (136.2; 308.0) |
| M24 |  | 33 | 188.2 (132.2; 268.0) | 34 | 229.0 (154.5; 339.6) |  | 30 | 176.8 (113.0; 276.5) | 35 | 181.2 (122.8; 267.3) |
| M36 |  | 32 | 215.7 (154.8; 300.5) | 34 | 254.4 (178.4; 362.6) |  | 29 | 193.3 (128.5; 290.9) | 35 | 174.2 (117.8; 257.6) |
| M48 |  | 32 | 233.0 (166.0; 327.0) | 31 | 278.4 (191.7; 404.3) |  | 29 | 168.1 (114.1; 247.6) | 33 | 159.8 (107.6; 237.4) |
| M60 |  | 33 | 209.7 (154.4; 284.7) | 34 | 224.1 (159.4; 315.2) |  | 30 | 189.8 (117.9; 305.6) | 35 | 180.3 (120.0; 270.7) |
| **HPV-070 study** | | | | | | | | | | |
| M7 | **HPV-31** | 90 | 1784.3 (1508.2; 2110.9) | 85 | 1241.1 (959.1; 1606.1) | **HPV-45** | 91 | 1909.8 (1577.5; 2312.0) | 86 | 1054.4 (855.6; 1299.3) |
| M12 |  | 90 | 310.0 (253.5; 379.3) | 85 | 319.02 (53.3; 401.6) |  | 91 | 485.1 (387.1; 608.0) | 86 | 335.6 (264.2; 426.3) |
| M18 |  | 89 | 197.5 (163.3; 238.8) | 85 | 213.8 (171.4; 266.8) |  | 90 | 282.1 (228.8; 347.7) | 86 | 214.4 (168.4; 272.8) |
| M24 |  | 90 | 165.1 (134.5; 202.6) | 85 | 188.4 (151.7; 234.1) |  | 91 | 221.4 (178.0; 275.5) | 86 | 194.4 (152.1; 248.3) |
| M36 |  | 90 | 148.3 (122.1; 180.0) | 85 | 156.3 (126.5; 193.2) |  | 91 | 175.3 (139.5; 220.2) | 86 | 168.7 (133.5; 213.3) |

2D, 2-dose schedule (M0,6) of the HPV-16/18 AS04-adjuvanted vaccine; 3D, 3-dose schedule (M0,1,6) of the HPV-16/18 AS04-adjuvanted vaccine; ATP-I, according-to-protocol immunogenicity cohort; 95% CI, two-sided 95% confidence intervals; ELISA, enzyme-linked immunosorbent assay; EU, enzyme-linked immunosorbent assay units; GMC, geometric mean concentration; HPV, human papillomavirus; N, number of subjects with pre-vaccination results available.

Supplementary Table 4. Observed CD4+ T-cell responses (in terms of median frequency of HPV-31/45 antigen-specific CD4+ T cells per million CD4+ T cells expressing at least two different immune markers [all doubles]) and B-cell responses (in terms of median frequency of HPV-31/45 antigen-specific memory B cells per million memory B cells in subjects with detectable B cells) at each timepoint for initially seronegative subjects in the HPV-070 (NCT01381575) M36 ATP-I cohort.

| Time-point | Antigen | 2D schedule  Girls 9-14 years | | 3D schedule  Women 15-25 years | | Antigen | 2D schedule  Girls 9-14 years | | 3D schedule  Women 15-25 years | |
| --- | --- | --- | --- | --- | --- | --- | --- | --- | --- | --- |
|  |  | N | Median frequency  (Q1; Q3) | N | Median frequency  (Q1; Q3) |  | N | Median frequency  (Q1; Q3) | N | Median frequency  (Q1; Q3) |
| **CD4+ T-cell response** | | | | | | | | | | |
| PRE | **HPV-31** | 82 | 86.5 (58.0; 149.0) | 75 | 141.0 (80.0; 226.0) | **HPV-45** | 83 | 85.0 (57.0; 132.0) | 77 | 110.0 (70.0; 157.0) |
| M7 |  | 71 | 2760.0 (1349.0; 5150.0) | 60 | 2209.5 (1109.5; 3620.5) |  | 71 | 2836.0 (1277.0; 4346.0) | 61 | 1635.0 (955.0; 2873.0) |
| M12 |  | 87 | 1794.0 (737.0; 3517.0) | 78 | 1462.0 (759.0; 2010.0) |  | 89 | 1948.0 (1052.0; 3418.0) | 81 | 1421.0 (778.0; 2303.0) |
| M24 |  | 80 | 1567.0 (839.0; 2759.5) | 73 | 1327.0 (668.0; 2101.0) |  | 81 | 1634.0 (1006.0; 3490.0) | 75 | 1543.0 (832.0; 2522.0) |
| M36 |  | 75 | 1324.0 (685.0; 2817.0) | 62 | 1101.5 (603.0; 1703.0) |  | 79 | 1678.0 (842.0; 3335.0) | 64 | 1349.0 (729.5; 2155.0) |
| **B-cell response** | | | | | | | | | | |
| PRE | **HPV-31** | 79 | 1.0 (1.0; 1.0) | 71 | 1.0 (1.0; 1.0) | **HPV-45** | 79 | 1.0 (1.0; 1.0) | 72 | 1.0 (1.0; 1.0) |
| M7 |  | 74 | 148.0 (1.0; 417.0) | 54 | 91.5 (1.0; 252.0) |  | 73 | 103.0 (1.0; 338.0) | 55 | 111.0 (18.0; 302.0) |
| M12 |  | 55 | 76.0 (1.0; 294.0) | 51 | 86.0 (1.0; 301.0) |  | 57 | 31.0 (1.0; 161.0) | 50 | 38.5 (1.0; 122.0) |
| M24 |  | 77 | 21.0 (1.0; 109.0) | 66 | 26.0 (1.0; 142.0) |  | 79 | 1.0 (1.0; 98.0) | 68 | 67.5 (1.0; 240.0) |
| M36 |  | 73 | 68.0 (1.0; 253.0) | 60 | 16.5 (1.0; 176.5) |  | 74 | 56.5 (1.0; 177.0) | 61 | 68.0 (1.0; 158.0) |

2D, 2-dose schedule (M0,6) of the HPV-16/18 AS04-adjuvanted vaccine; 3D, 3-dose schedule (M0,1,6) of the HPV-16/18 AS04-adjuvanted vaccine; ATP-I, according-to-protocol immunogenicity cohort; N, number of subjects with pre-vaccination status available; M, month; PRE, pre-vaccination; Q1, 25% percentile; Q3, 75% percentile.

Supplementary Table 5. Observed HPV-16 and HPV-18 GMC by ELISA at each timepoint for initially seronegative subjects in the M36 (HPV-070; NCT01381575) or M60 (HPV-048; NCT00541970) ATP-I cohort.

| Time-point | Antigen | 2D schedule  Girls 9-14 years | | 3D schedule  Women 15-25 years | | Antigen | 2D schedule  Girls 9-14 years | | 3D schedule  Women 15-25 years | |
| --- | --- | --- | --- | --- | --- | --- | --- | --- | --- | --- |
|  |  | N | GMC, (95% CI) | N | GMC, (95% CI) |  | N | GMC, (95% CI) | N | GMC, (95% CI) |
| **HPV-048 study^a^** | | | | | | | | | | |
| M7 | **HPV-16** | 45 | 12444.6 (9930.2; 15595.7) | 79 | 11492.0 (9059.4; 14577.7) | **HPV-18** | 43 | 5880.7 (4721.3; 7324.8) | 76 | 4511.6 (3658.0; 5564.4) |
| M12 |  | 45 | 3717.7 (2990.1; 4622.4) | 78 | 3970.6 (3116.5; 5058.7) |  | 43 | 1693.8 (1271.5; 2256.4) | 75 | 1535.3 (1202.7; 1959.9) |
| M18 |  | 45 | 2465.0 (1974.8; 3076.7) | 77 | 2559.6 (2022.4; 3239.5) |  | 43 | 976.1 (724.3; 1315.5) | 74 | 1033.6 (801.1; 1333.5) |
| M24 |  | 45 | 1857.9 (1500.6; 2300.2) | 78 | 2050.0 (1630.7; 2576.9) |  | 43 | 745.2 (554.2; 1002.1) | 75 | 816.7 (634.4; 1051.4) |
| M36 |  | 43 | 1654.3 (1316.8; 2078.2) | 79 | 1735.5 (1393.4; 2161.7) |  | 41 | 705.4 (516.3; 963.8) | 76 | 732.7 (564.5; 951.2) |
| M48 |  | 44 | 1374.1 (1111.6; 1698.7) | 74 | 1466.1 (1172.4; 1833.3) |  | 42 | 566.6 (428.0; 749.9) | 73 | 598.5 (460.5; 777.8) |
| M60 |  | 45 | 1369.0 (1104.0; 1697.5) | 79 | 1454.5 (1187.2; 1782.1) |  | 43 | 627.2 (476.1; 826.1) | 76 | 598.5 (460.5; 777.8) |
| **HPV-070 study^b^** | | | | | | | | | | |
| M7 | **HPV-16** | 455 | 9402.9 (8792.4; 10055.8) | 330 | 10120.2 (9162.7; 11177.9) | **HPV-18** | 462 | 5935.6 (5519.4; 6383.3) | 356 | 4984.2 (4543.9; 5467.1) |
| M12 |  | 455 | 2653.5 (2473.5; 2846.6) | 330 | 3290.4 (2956.5; 3662.0) |  | 462 | 1523.6 (1403.7; 1653.7) | 356 | 1491.5 (1339.0; 1661.4) |
| M18 |  | 453 | 1730.7 (1608.6; 1862.0) | 329 | 1931.2 (1735.4; 2149.1) |  | 459 | 864.6 (793.1; 942.7) | 355 | 830.6 (742.9; 928.5) |
| M24 |  | 454 | 1483.8 (1382.1; 1592.9) | 326 | 1575.9 (1418.2; 1751.2) |  | 460 | 715.5 (658.1; 777.9) | 352 | 654.3 (582.9; 734.5) |
| M36 |  | 455 | 1210.2 (1124.8; 1302.1) | 330 | 1326.4 (1193.9; 1473.5) |  | 462 | 562.8 (516.4; 613.4) | 356 | 552.6 (494.1; 618.0) |

2D, 2-dose schedule (M0,6) of the HPV-16/18 AS04-adjuvanted vaccine; 3D, 3-dose schedule (M0,1,6) of the HPV-16/18 AS04-adjuvanted vaccine; ATP-I, according-to-protocol immunogenicity cohort; 95% CI, two-sided 95% confidence intervals; ELISA, enzyme-linked immunosorbent assay; EU, enzyme-linked immunosorbent assay units; GMC, geometric mean concentration; HPV, human papillomavirus; N, number of subjects with pre-vaccination results available.

^a^Data from Barbara Romanowski,Tino F. Schwarz, Linda Ferguson, et al. Sustained Immunogenicity of the HPV-16/18 AS04-Adjuvanted Vaccine Administered as a Two-Dose Schedule in Adolescent Girls: Five-Year Clinical Data and Modelling Predictions from a randomized study. Hum Vaccin Immunother**. 2016**;2:20-29.

^b^Data from Huang LM, Puthanakit T, Chiu CH, et al. Sustained Immunogenicity of 2-dose Human Papillomavirus 16/18 AS04-adjuvanted Vaccine Schedules in Girls Aged 9–14 Years: A Randomized Trial. J Infect Dis. **2017**;215:1711-1719.
