# Supplementary material for: Long-term Cross-reactivity Against Nonvaccine Human Papillomavirus Types 31 and 45 After 2- or 3-Dose Schedules of the AS04-Adjuvanted Human HPV-16/18 Vaccine
Source: J Infect Dis. 2019 Feb 3;219(11):1799–803. doi: 10.1093/infdis/jiy743 (PMC6500548; doi:10.1093/infdis/jiy743)
Supplement: jiy743_suppl_Supplementary_materials [file jiy743_suppl_supplementary_materials.docx]

**Focus on the Patient Section:**

- HPV-16 and -18 are associated with 70% of cervical cancers, while HPV-31 and -45 contribute to an additional 10%.
- The AS04-HPV-16/18 vaccine showed similar immunogenicity against HPV-16/18 in girls receiving 2 doses compared to women receiving 3 doses.
- Here we showed that cross-reactivity of the vaccine against HPV-31/45 is also similar after 2 or 3 doses and lasts for at least 5 years, supporting long-lasting cross-protection against the same HPV types.

**Data sharing statement**

Anonymized individual participant data and study documents can be requested for further research from [www.clinicalstudydatarequest.com](http://www.clinicalstudydatarequest.com/).
